# Improving Outcomes with Surgical Resection and Other Ablative Therapies in HCC

**DOI:** 10.4061/2011/686074

**Published:** 2011-06-12

**Authors:** Rahul Deshpande, Derek O'Reilly, David Sherlock

**Affiliations:** Department of Hepatobiliary Surgery, North Manchester General Hospital, Crumpsall, Manchester M8 5RB, UK

## Abstract

With rising incidence and emergence of effective treatment options, the management of hepatocellular carcinoma (HCC) is a complex multidisciplinary process. There is still little consensus and uniformity about clinicopathological staging systems. Resection and liver transplantation have been the cornerstone of curative surgical treatments with recent emergence of ablative techniques. Improvements in diagnostics, surgical techniques, and postoperative care have lead to dramatically improved results over the years. The most appropriate treatment plan has to be individualised and depends on a variety of patient and tumour-related factors. Very small HCCs discovered on surveillance have the best outcomes. Patients with advanced cirrhosis and tumours within Milan criteria should be offered transplantation. Resection is best for small solitary tumours with preserved liver function. Ablative techniques are suitable for low volume tumours in patients unfit for either resection or transplantation. The role of downstaging and bridging therapy is not clearly established.

## 1. Introduction

Hepatocellular carcinoma (HCC) is the fifth commonest cancer in the world in men and is the third highest contributor towards any cancer-related mortality [1]. Although the highest number of cases relates to the Far East, Middle-East, and Africa, the last decades have seen nearly a doubling of the incidence of HCC in the western world, particularly in the United States with the trend still on the rise [2]. The vast majority of HCCs occur on a background of pre-existing liver disease, and more recently, Hepatitis C (HCV) and nonalcoholic steatohepatitis (NASH) have emerged as the leading causes of cirrhosis and thereby, HCC. 

Unfortunately, late presentation is all too common, and a recent analysis of the SEER (Surveillance, Epidemiology and End Result) database revealed that just about 16% of patients were suitable for some kind of surgical therapy in the form of resection, transplantation, or ablation [3]. However, surgical and ablative modalities offer the only possibility of long-term cure. Surgical treatment options depend not only on the extent of the cancer but also on the status of the liver and the general condition of the patient. In theory, the best outcomes should be obtained in patients with well-compensated liver disease without severe portal hypertension, who are generally in good health and have a small tumour burden. Hence surveillance programmes have been established, particularly in high-risk geographical areas, for the early detection of HCCs to facilitate curative treatment and improved outcomes [4]. This paper discusses the improving outcomes associated with surgical management of HCC. 

## 2. Staging of HCC

Clinical management of HCC is a multidisciplinary process involving hepatologists, surgeons, oncologists, and radiologists. Staging is an integral part of the decision-making process so that treatment can be individualised. Since the vast majority of HCCs occur on a background of liver disease with or without cirrhosis, it is clear that prognosis does not depend exclusively on tumour-related factors but also on liver function, general health, and other comorbidities of the patient and response to various medical treatments depending on the aetiology [5]. Staging of HCC is difficult, and a wide variety of systems have been in existence for years. They include those that deal exclusively with tumour staging pre- and postoperatively, pathological staging of the resected tumour, general staging for liver disease, and those that were initially developed to predict factors determining outcome of liver disease with or without HCC. However, only three of them have been validated in patient cohorts [6]. The detailed description and analysis of these are beyond the scope of this paper. The current consensus is that no single staging system is universally applicable and there is significant heterogeneity in patient groups even within the same stage, particularly since the aetiology and prognosis of liver disease are multifold [7]. Tumour size alone is an inconsistent determining factor, yet transplant criteria are based predominantly on size. Histological characteristics which can potentially prognosticate outcome cannot unfortunately be determined preoperatively. Some uniformity in staging is essential, and the various diagnostic tools used to stage and assess HCC pretreatment need to be standardised. 

The BCLC (Barcelona Clinic Liver Cancer) staging, which has been recently updated, attempts to match staging with both prognosis and treatment options, thereby developing an evidence-based algorithm for the surgical and medical management of HCC [8]. This has not gained universal acceptance and is probably popular mainly in the European countries. Other geographical study groups have also published consensus recommendations. The Asian Pacific Association for the Study of Liver (APASL) convened an international working party to develop consensus recommendations for the diagnosis, surveillance, and management of HCC. A treatment algorithm was proposed, which, again, takes into account resectability and size of the HCC and the general condition of the patient [9]. Finally, the National Comprehensive Cancer Network (NCCN) has published and updated clinical practice guidelines for hepatobiliary oncology with a treatment algorithm for the diagnosis and management of HCC which, similar to the APASL, is based on resectability, status of liver disease, and general condition [10]. Unlike the BCLC, neither specifically makes an attempt to formally classify HCC into stages. It should, however, be noted that both the APASL and the NCCN are consensus guidelines derived from evidence-base and large panels of international experts rather than a single-centre approach. Individual aspects of these algorithms are discussed further in the paper. 

## 3. Surgical Modalities for the Management of HCC

### 3.1. Liver Transplantation

Liver transplantation (LT) has been used to treat HCC with cirrhosis for nearly three decades. It is the only surgical treatment which simultaneously addresses the liver condition and, essentially, cures two related problems. Early results of LT for HCC were disappointing. In absence of specific inclusion criteria, many patients with HCC were transplanted for advanced disease resulting in an overall poor survival [11–13]. For LT to be universally acceptable as a treatment option for HCC, it not only had to demonstrate better results than other treatment options but also to achieve at least 50% survival at 5 years to justify use of scarce donor organs. Therefore, it was necessary that certain criteria are used to preselect patients suitable for transplantation. Increasing tumour size and bulk, vascular invasion, extrahepatic nodal disease, and worse tumour histology were long known to be indicators of poor prognosis with other treatment modalities. Prognosis after transplantation for HCC was also noted to be better in early stage disease. It was only in 1996 that Mazzaferro et al. in a prospective study clearly defined inclusion criteria (single tumour <5 cm or 1–3 tumours, none >3 cm) which showed a significant survival benefit [14]. Since then, the so-called “Milan criteria” have gained universal acceptance, and the results have been replicated in other studies [15, 16]. These have long been adopted by UNOS (United network for organ sharing) as the optimal criteria for LT and in the TNM staging. After the introduction of MELD score for organ allocation, bonus “points” were awarded to early stage (T1 and T2) HCCs in an attempt not to disadvantage patients with low-grade tumours and good synthetic liver function. However, this led to an overcorrection and a significant increase in the number of patients transplanted for HCCs at the expense of other indications. It was noted that the dropout rate for T1 tumours in the pre-MELD era was under 10%, which is less than the overall waiting list mortality. This has thus led to elimination of the score upgrading for T1 lesions and a lesser upgrade for T2 lesions [17, 18]. This has not necessarily had an adverse impact on survival, and there has been a significant increase in the number of transplants performed for early HCC in cirrhosis [19]. 

#### 3.1.1. Results of Liver Transplantation for HCC

The original Milan study reported 4-year and recurrence-free survival rates of 75% and 83%, respectively, and their 10-year overall survival is over 70% in transplants performed for HCC within Milan criteria [20]. Similar results have been achieved in other centres, and a 5-year survival of well over 70% has been reported in patients undergoing LT for HCC within Milan criteria [15, 16]. 

It has to be argued, however, that these are data from single centres and may not accurately reflect the entire picture. Any comparison between differing treatment modalities has to take into account an intention-to-treat analysis which, in case of LT, is tempered by waiting times and waiting list dropout and mortality which vary between 20% and 30% [4]. It has been shown that mortality from the time of listing for LT increases significantly with increasing waiting times [21]. Pooled registry data incorporating a very large number of patients have clearly shown that the long-term survival figures do not necessarily replicate data from the best single centres. An analysis of 4482 patients within the UNOS Organ Procurement Transplant Network data demonstrated that overall intention-to-treat 5-year survival after LT for HCC was 61% even for those favourable group of patients within Milan criteria [22]. This figure went up to 65% when only patients who underwent LT were taken into account.  Table 1 summarises outcomes after LT for HCC in major series.

Over the last decade, there has been a vigorous debate over expansion of the Milan criteria. Proponents have demonstrated outcomes comparable to those with Milan criteria and argue that maintaining the restriction of criteria would exclude patients that would otherwise do well after transplantation despite a larger tumour burden [23–25]. Others equally insist that original criteria are strictly adhered to in view of the inconsistent results achieved after transplanting patients with larger tumours, specifically arguing that potential beneficiaries of expansion of criteria have significantly worse outcomes [26]. The UNOS data demonstrated that the intention-to-treat 5-year survival for patients listed LT for HCC beyond Milan criteria was only 32% and 38% for those patients that actually underwent LT [22].  Table 2 summarises results of these studies. Retrospective designs of most studies and the impossibility of prospective randomised controlled comparative studies between these groups make direct comparisons unreliable. Also, there is no definite consensus in terms of how further should these criteria be expanded. Finally, ready acceptance to expand the criteria is tempered by the two critical issues facing the transplantation community: burgeoning waiting times and increasing dropout rates and mortality due to tumour progression and complications of underlying liver disease. Hence, currently, Milan criteria still remain valid in most transplant units. 

#### 3.1.2. Factors Determining Outcome after LT for HCC

There is clear evidence that postoperative histology correlates with disease recurrence and survival. Even in the group of patients within Milan criteria, vascular invasion and tumour undifferentiation carry worse prognosis [15, 27]. Others have demonstrated that variables, such as higher total tumour burden, higher preoperative alpha-fetoprotein, and presence of tumour necrosis, predict significantly worse outcomes [28, 29]. It has been discussed that survival of patients beyond Milan but within UCSF criteria is worse thereby suggesting that tumour size may indeed be a surrogate marker of adverse histological features such as vascular invasion, the one criterion consistently shown to predict worse survival. 

#### 3.1.3. Living Donor Liver Transplantation (LDLT) for HCC

Dropout rate for HCC is related both to waiting list mortality in patients with advanced liver disease per se and to tumour progression beyond Milan criteria despite preserved liver function. LDLT could potentially benefit such patients who would otherwise be rendered nontransplantable. It could also cater for the 10–20% patients with HCC beyond Milan but within UCSF criteria without having an impact on the availability and distribution of organs across the program. Patient survival and disease-free survival after LDLT for HCC are comparable to cadaveric transplantation. As with other series, tumour size, histological grade, vascular permeation, preoperative serum alpha-fetoprotein, and preoperative MELD score correlated with survival and disease recurrence. Survival was worse in patients beyond Milan criteria [30, 31]. In another study, LDLT had poorer outcome compared to cadaveric transplantation in patients with large HCCs [32]. Although it was not statistically significant, the 2-year patient survival was 60%. Long-term outcomes were not reported.

The ethical issues with LDLT and, in particular, the potential dangers to a healthy adult have been long debated. There is small but significant risk mortality to the donor. Significant morbidity occurs in about 20% of donors, and up to 50% experience various minor complications [33]. Postoperative biliary and vascular complications are higher in the recipient. The projected patient and graft survival rates are potentially lower in view of these complications and LDLT being used for more advanced disease. HCV-associated cirrhosis, which is frequently associated with HCC, carries a worse prognosis, and there is evidence that recurrence is more severe after LDLT [34]. There is a danger that if LDLT is advocated for tumours beyond Milan criteria, there would be a pressure on the patient to find a suitable living donor and on the donor to fulfil an obligation for a potentially nonbeneficial cause. Altruistic donation, too, could come under close scrutiny if used for such indications. However, HCC represents a good indication for LDLT. The majority of patients listed for LT do not have advanced liver disease and would have a much better prognosis than patients transplanted for end-stage liver disease. 

Families involved in LDLT need to be given clear advice, and their wishes need to be taken into consideration. Since LDLT does not primarily tap into cadaveric organ pool, even a slightly low survival should be considered acceptable since the outcome would be significantly better than other treatment modalities. However, in the event of graft failure in a patient transplanted for HCC exceeding current criteria, it would be highly contentious to turn to retransplantation with a cadaveric organ, and this issue has to be clearly discussed with the family. Thus LDLT has an important role to fill in LT for HCC. 

### 3.2. Surgical Resection

Surgical resection for HCC was the only modality for curative treatment before the routine use of LT and has several practical advantages over it. Firstly, there are no restrictions on tumour size and numbers as long as resection can be safely performed. Secondly, resections can be performed in any nontransplant setup that has an adequate radiological and medical backup. Finally, it does not rely on a donor pool, and there are no waiting times for treatment. However, there are distinct disadvantages. Resection does not address and cure the background liver disease, thereby retaining a fertile background for recurrence. Secondly, it compromises an already damaged liver by removing vital functioning liver mass and is reliant on a well functioning liver with adequate reserves. Finally, in the event of liver decompensation and failure, emergency liver transplantation as a rescue treatment may not be available, particularly if the Milan criteria in the resected specimen have been exceeded or if poor prognostic features are evident on histology. 

#### 3.2.1. Results for Liver Resection for HCC

The key factor that determines outcomes is patient selection. As mentioned before, although tumour bulk need not necessarily present restriction, liver function and extent of the background liver disease do. Just as early results with LT for HCC were disappointing, so were results with resection, and the best 5-year survival rates in the 1980s were about 32% [35]. These figures were significantly improved upon in the later decades with large Eastern studies demonstrating a 5-year survival of nearly 50% in the latter part of the series which compared favourably with 36% seen in the earlier cohort [36]. Interestingly, it was also shown that significantly more patients in the latter group were found to have HCCs at an earlier stage, and many were detected at a subclinical stage. This was clearly a result of emerging surveillance programmes. Recently, median survival rates of 75 months and 5-year survival rates of 64% to 70% have been reported which compare favourably with results with LT [37, 38]. Latest series from the far east have demonstrated a very high 5-year survival rate of 79–81% in patients with early HCC up to 5 cm although the majority of patients in this series had favourable background factors [39, 40]. However, even in expert centres, results are significantly worse for larger HCCs, especially those exceeding Milan criteria [41]. Once again, it has to be acknowledged that these are data from high-volume centres in a select group of patients and may not reflect the overall picture. Despite these excellent results, recurrence rates at 5 years, even after curative resections, remain disappointingly high at nearly 80% with early recurrence observed in nearly a third of patients [37, 42]. Rates of early and cancer-related deaths are relatively high at 8–10%, particularly for extended resections [43]. Outcomes are particularly poor in resection performed for advanced HCC with multinodular or microvascular involvement [44].  Table 3 summarises results seen with resection for HCCs and clearly demonstrates significant improvement in survival figures over the last 10 years. 

#### 3.2.2. Factors Determining Outcome after Resection for HCC

With background liver disease remaining untreated, postoperative morbidity and noncancer mortality are related to the extent of liver dysfunction, presence of significant portal hypertension, and intraoperative factors such as blood loss. An attempt must be made to spare as much liver parenchyma as possible.

Specific tumour-related factors have been analysed by many authors in an attempt to prognosticate outcome and also to streamline patient selection. Multiple tumours, vascular invasion, preoperative serum alpha-fetoprotein levels, and tumour size are independent predictors of outcome [37]. Similar factors along with extent of mitoses were demonstrated to significantly determine outcome in a multicentre study [45]. This study also demonstrated a high 1-year mortality of 22%, the majority of them dying of cancer recurrence or liver failure. In solitary HCCs, where parenchymal sparing is not necessarily a problem, a wide surgical resection margin of 2 cm is associated with lower rates of recurrence and better outcomes [46]. There is also some recent evidence that patients undergoing resection for small HCCs associated with cirrhosis due to HBV have better long-term survival than those with HCV [47]. This is relevant whilst interpreting and comparing data from differing geographical areas since it is evident that the majority of patients in the far east have cirrhosis related to HBV infection whereas the commonest aetiology in the western world is HCV. Large multifocal HCCs with vascular involvement should be considered as contraindications for major liver resections as should association of three or more of the other risk factors [37, 44]. Thus, although there are no current restrictions on the upper limit of size of HCC suitable for resection, it is clear from these data that best results are, once again, obtained in solitary or small HCCs confirming to Milan criteria. 

#### 3.2.3. Preoperative Portal Vein Embolisation (PVE) for Major Resections for HCC

Portal vein embolisation is a well-established method to increase the volume and function of the future liver remnant (FLR) prior to major liver resection for any pathology. The number of patients that undergo hypertrophy after PVE and the extent of that hypertrophy is less in livers with chronic liver disease compared to that seen in normal livers [48]. A large meta-analysis has confirmed the safety and efficacy of PVE with low morbidity and ability to perform major resections with very low mortality [49]. Another study has demonstrated better immediate outcomes in liver resections performed for HCC with PVE than without [50]. Unlike normal livers, the FLR necessary for cirrhotic livers is purported to be up to 40% even in presence of preserved liver function [51]. Recently, PVE has also been used in combination with transarterial chemoembolisation (TACE) to demonstrate better hypertrophy, postoperative outcomes, and recurrence-free survival compared to PVE alone, although no prospective randomised trials exist [52]. Thus, on the current evidence, PVE is safe and efficacious and should be regularly used to enable major curative resections for HCC. 

#### 3.2.4. Resection or Transplantation for HCC? 

With comparable results achievable with either LT or resection in select patient groups, there has been a vigorous debate in terms of the best possible curative option for HCC. The pros and cons of both modalities have already been outlined in this paper. It is clear that best results are achieved in small HCCs, typically within Milan criteria, with excellent liver function and no associated comorbidities. More recently, comparable results were demonstrated between heterogeneous groups of patients undergoing LT or resection for HCCs beyond Milan criteria although the median followup was only 34 months and significantly more patients in the LT group had established cirrhosis [41]. Outcomes have to be based on an intention-to-treat basis, taking into account waiting times, dropouts, and waiting-list mortality. It would be impossible to perform a prospective randomised trial to establish the best modality of treatment. Although LT addresses the issue of liver disease, recurrence for certain conditions such as the viral hepatides is common. These patients also have to be on life-long immunosuppression which is unnecessary after a liver resection. 

A recent consensus conference concluded that LT is the preferred method for patients with cirrhosis and HCC meeting Milan criteria while resection with wide margins is the treatment of choice for selected patients with cirrhosis that have well-preserved liver function, with no portal hypertension without a size restriction [53]. The Barcelona group have also proposed an algorithm based on their staging system and suggest that resection should be used for very early single HCCs (<2 cm) with normal liver function and no portal hypertension whereas all other patients within Milan criteria and suitable for curative treatment should be considered for LT [8]. The APASL guidelines recommend liver resection as a first-line curative treatment of solitary or multifocal HCC confined to the liver, which are anatomically resectable and where there is satisfactory liver function reserve. LT should preferably be used for HCCs within Milan criteria in patients with more advanced liver disease (Child Pugh B or C) if medically fit [9]. The NCCN guidelines are similar but more ambiguous about the optimal size of HCC best suited for resection. LT is reserved for patients within Milan criteria and more advanced liver disease and potentially for those that are “unresectable” due to unfavourable tumour location or inadequate liver reserve [10]. This reflects the increasing use of resection as the first line of management for early HCC and may, in part, be also due to the fact that UNOS criteria specify that patients eligible for LT should not be considered for resection. Thus, although there are certain differences in all these guidelines, it is clear that use of surgical resection as the first treatment for small HCCs in a well-preserved liver is increasingly prevalent. This is clearly helped by the fact that although long-term results after LT have remained relatively static over the last 10 years, those with resection have significantly improved. 

### 3.3. Ablative Techniques for HCC

Ablative techniques have an established role in the management of HCC. Radiofrequency ablation (RFA) is the most commonly used ablative technique for HCC. Other modalities include percutaneous ethanol injection (PEI) and microwave ablation (MWA). RFA is a minimally invasive procedure that can be performed percutaneously or operatively using both an open and a laparoscopic approach, with relatively low major complication rates. It can also be performed in patients who would be unsuitable for surgery (either LT or resection) due to associated comorbidity. It, however, is the only potentially curative technique that does not permit histological analysis of the tumour, and hence tumour-based prognostic criteria for best outcomes cannot be readily determined. In terms of safety and efficacy, a large meta-analysis in 2001 demonstrated an overall mortality of 0.5% and a complication rate of 8.9% [54]. More recent studies have demonstrated further lowering of mortality (0.1%) and complication rates [55]. The commonest complications are liver abscesses, biliomas, haemorrhage, and so forth. Although both RFA and PEI are effective techniques, studies have demonstrated that necrotic effect of RFA is more predictable for larger tumour sizes and that RFA is superior in terms of local tumour progression and disease-free survival [56]. 

#### 3.3.1. Results of RFA for HCC

RFA is best used for tumours less than 3 cm after which the incidence of local recurrence increases. Vascular proximity leads to a heat-sink effect minimising the efficacy of the burn and thereby promoting higher recurrence rates. Significantly better results seem to be obtained when the procedure is performed operatively rather than percutaneously [57]. A randomised trial reported comparable outcomes between RFA and surgical resection for solitary small HCCs <5 cm with 4-year survival rates between 64 and 68% [58]. Another more recent study demonstrated sustained complete response rate for RFA for very small HCCs to be over 97% with a 5-year survival of 69% in tumours that would be considered operable [59]. This was improved upon in another series reporting a 5-year survival rate of 76% for patients considered operable disease by BCLC criteria [60]. A large retrospective study demonstrated that in Child Pugh A cirrhotics, RFA and resection offered equivalent benefits for tumours less than 3 cm while resection provided better survival when the HCC was larger than 3 cm but still within Milan criteria [61]. A Chinese study demonstrated 5- and 7-year survival of 60% and 55% with RFA as the primary treatment for HCCs within Milan criteria [62]. The latest randomised controlled trial comparing RFA and resection for HCCs within Milan criteria demonstrated that overall survival and recurrence-free survival were significantly better with curative resection rather than RFA [63]. In this study, the overall 5-year survival rates were 55% with RFA and 76% with resection, both comparing favourably with LT. Most of these series enrolled patients with Child Pugh score A and tumours either within Milan criteria or small solitary tumours <5 cm.  Table 4 summarises results of RFA for HCCs.

These data would suggest that RFA is probably as effective as both resection and LT for small HCCs in early cirrhotic patients with preserved liver function. However, more prospective randomised trials with much larger number of patients would be necessary to demonstrate the superior treatment modality. Recent trials have demonstrated slight inferiority of RFA over resection for small HCCs. The BCLC algorithm recommends RFA as the primary treatment for single small HCCs in patients that are high risk for operative management due to associated comorbidity [8]. Similar approach is advocated by both APASL and the NCCN which recommend RFA as an equivalent alternative for any HCC considered suitable for resection, namely, solitary HCC <3 cm in a patient with Child Pugh A cirrhosis. This approach may yet change as more data on randomised trials between RFA and resection becomes available.

More recently, other ablative techniques such as MWA and high-intensity focussed ultrasound (HIFU) have been used in an attempt to overcome some of the limitations of RFA. These and others such as electroporation need to be further evaluated in clinical trials [64]. 

#### 3.3.2. RFA as a Bridge or a Holding Therapy to Transplantation

Longer waiting times for LT and rising dropout rates prompted the evaluation of RFA and TACE as holding techniques for patients who would otherwise progress beyond Milan criteria and also for downstaging patients with HCCs already beyond Milan criteria to make them eligible for LT [65]. Such early feasibility studies indicated that some tumours could be successfully downstaged. However, it is doubtful whether downstaging larger tumours with RFA or TACE alters prognosis or biological behaviour of the tumour. A study which applied this practice demonstrated overall 5-year survival less than 50% [66]. In fact, nearly half of all patients “dropped out,” and the survival on an intention-to-treat basis was less than 25%. It is assumed that nonresponders are a self-selecting group of patients that manifest unfavourable tumour biology. Much better outcomes were demonstrated recently with a dropout rate of 30% and an intention-to-treat 4-year survival of 69% [67]. 

The role of RFA and TACE as holding therapy for patients satisfying organ allocation criteria is equally contentious. It could be employed to minimise dropout rates, a natural occurrence on waiting lists. However, it does not confer additional benefits or improve survival after successful LT [68]. Once again, large randomised trials are necessary to conclusively demonstrate benefit but are probably not feasible. 

## 4. Conclusions

HCC is a common but complex multifactorial condition with poor outcomes worldwide. Few patients ever come to curative surgical therapy. The role of surgical techniques has gradually expanded over the years with significant improvement in outcomes with liver resection whilst those with LT have remained static. Outcome after any curative treatment for HCC is related not only to the stage of the tumour but also to tumour biology, background liver disease, and associated comorbidities.

Staging algorithms such as those proposed by the BCLC, APASL, and the NCCN provide useful, evidence-based guidance towards decision-making. Treatment should be individualised, preferably in high-volume centres of expertise with facilities for both liver resection and liver transplantation. 

For small solitary HCCs <2-3 cm and normal liver function, LT, resection and RFA seem to confer comparable benefit although recent trials confirm superiority of resection compared to RFA. Milan criteria continue to be utilised by most transplant programs, and although there seems to be a case for modest increase, the results have not been replicated across national programs. LT should be the preferred approach for patients with larger tumours within Milan criteria especially with advanced liver dysfunction. Resection should be preferred either for very small HCCs or for those that have exceeded Milan criteria but still have excellent liver function and no portal hypertension. RFA should be the preferred method of ablation and should be utilised for small HCCs not otherwise fit for surgical management. 

## Figures and Tables

**Table 1:** Results of liver transplantation for HCC.

Author	Year	Number of patients	Inclusion criteria if any	3-year survival (%)	5-year survival (%)
Iwatsuki et al. [11]	1991	105	None	47	
Bismuth et al. [12]	1993	60	None	47	
Ringe et al. [13]	1991	61	None		15.2
Mazzaferro et al. [14]	1996	48	Milan criteria		74 (4-year)
Jonas et al. [15]	2001	120	Milan		71
Figueras et al. [16]	2001	307	Milan		63
Yao et al. [23]	2001	70	USCF		75^a^
Onaca et al. [24]	2007	1206	Milan		62
Duffy et al. [25]	2007	467	Milan UCSF		79 64^b^
Pelletier et al. [22]	2009	2898	Milan		65^c^
Cescon et al. [29]	2010	283	Milan		75

UCSF: University of California San Francisco criteria (single tumour <6.5 cm, 2-3 tumours, none >4.5 cm and total tumour dimensions up to 8 cm).

^a^Survival for all patients within UCSF criteria.

^b^Survival for patients beyond Milan but within UCSF criteria.

^c^Intention-to-treat survival: 61%.

**Table 2:** Results of liver transplantation for HCC beyond Milan criteria, based on preoperative imaging. (From national/large regional studies.)

Author	Year	Number of patients	5-year survival (%)	Notes
Decaens et al. [26]	2006	44 145	45.6 34.7	Beyond Milan, within UCSF Beyond UCSF
Duffy et al. [25]	2007	185 109	64 41	Beyond Milan, within UCSF Beyond UCSF
Pelletier et al. [22]	2009	346	38 32	Beyond Milan Intention-to-treat survival

**Table 3:** Improving results of liver resection for HCC.

Author	Year	Number of patients	Inclusion criteria if any	3-year survival (%)	5-year survival (%)
Iwatsuki and Starzl [35]	1988	55	None		25
Poon et al. [36]	2001	136^a^ 241^b^	None	47 62	36 49
Shi et al. [46]	2007	169	Solitary HCC^c^	79	61
Ishii et al. [38]	2008	162	Milan	89	70
Yamakado et al. [40]	2008	62	Milan	93	81
Canter et al. [41]	2010	94	Exceeding Milan	66	
Huang et al. [63]	2010	115	Milan	92	76
Hung et al. [39]	2011	229	Milan		79.3

^a^Resections performed between 1989 and 1994.

^b^Resections performed between 1994 and 1999.

^c^Majority within Milan criteria.

**Table 4:** Results for RFA for HCC.

Author	Year	Number of patients	Inclusion criteria if any	3-year survival (%)	5-year survival (%)
Chen et al. [58]	2006	71	Solitary <5 cm	71	68^a^
Livraghi et al. [59]	2008	218	Solitary <2 cm	76	55^b^
N'Kontchou et al. [60]	2009	222	Up to 3 HCC <5 cm		40^c^
Peng et al. [62]	2010	224	Solitary <5 cm		60
Huang et al. [63]	2010	115	Milan	70	55

^a^4-year survival.

^b^Survival increased to 69% for “operable” patients.

^c^Survival increased to 76% for “operable” patients.

## References

[1] Bosch FX, Ribes J, Cléries R, Díaz M (2005). Epidemiology of hepatocellular carcinoma. *Clinics in Liver Disease*.

[2] El-Serag HB, Mason AC (1999). Rising incidence of hepatocellular carcinoma in the United States. *New England Journal of Medicine*.

[3] Schwarz RE, Smith DD (2008). Trends in local therapy for hepatocellular carcinoma and survival outcomes in the US population. *American Journal of Surgery*.

[4] Bruix J, Sherman M (2005). Management of hepatocellular carcinoma. *Hepatology*.

[5] Okuda K (2002). Natural history of hepatocellular carcinoma including fibrolamellar and hepato-cholangiocarcinoma variants. *Journal of Gastroenterology and Hepatology*.

[6] Pons F, Varela M, Llovet JM (2005). Staging systems in hepatocellular carcinoma. *HPB*.

[7] Vauthey JN, Dixon E, Abdalla EK (2010). Pretreatment assessment of hepatocellular carcinoma: expert consensus statement. *HPB*.

[8] Forner A, Reig ME, de Lope CR, Bruix J (2010). Current strategy for staging and treatment: the BCLC update and future prospects. *Seminars in Liver Disease*.

[9] Omata M, Lesmana LA, Tateishi R (2010). Asian Pacific Association for the Study of the Liver consensus recommendations on hepatocellular carcinoma. *Hepatology International*.

[10] Benson AB, Abrams TA, Ben-Josef E (2009). NCCN clinical practice guidelines in oncology: hepatobiliary cancers. *Journal of the National Comprehensive Cancer Network*.

[11] Iwatsuki S, Starzl TE, Sheahan DG (1991). Hepatic resection versus transplantation for hepatocellular carcinoma. *Annals of Surgery*.

[12] Bismuth H, Chiche L, Adam R, Castaing D, Diamond T, Dennison A (1993). Liver resection versus transplantation for hepatocellular carcinoma in cirrhotic patients. *Annals of Surgery*.

[13] Ringe B, Pichlmayr R, Wittekind C, Tusch G (1991). Surgical treatment of hepatocellular carcinoma: experience with liver resection and transplantation in 198 patients. *World Journal of Surgery*.

[14] Mazzaferro V, Regalia E, Doci R (1996). Liver transplantation for the treatment of small hepatocellular carcinomas in patients with cirrhosis. *New England Journal of Medicine*.

[15] Jonas S, Bechstein WO, Steinmüller T (2001). Vascular invasion and histopathologic grading determine outcome after liver transplantation for hepatocellular carcinoma in cirrhosis. *Hepatology*.

[16] Figueras J, Ibañez L, Ramos E (2001). Selection criteria for liver transplantation in early-stage hepatocellular carcinoma with cirrhosis: results of a multicenter study. *Liver Transplantation*.

[17] Marsh JW, Dvorchik I (2003). Liver organ allocation for hepatocellular carcinoma: are we sure?. *Liver Transplantation*.

[18] Roayaie K, Feng S (2007). Allocation policy for hepatocellular carcinoma in the MELD era: room for improvement?. *Liver Transplantation*.

[19] Sharma P, Harper AM, Hernandez JL (2006). Reduced priority MELD score for hepatocellular carcinoma does not adversely impact candidate survival awaiting liver transplantation. *American Journal of Transplantation*.

[20] Mazzaferro V, Chun YS, Poon RT (2008). Liver transplantation for hepatocellular carcinoma. *Annals of Surgical Oncology*.

[21] Llovet JM, Fuster J, Bruix J (1999). Intention-to-treat analysis of surgical treatment for early hepatocellular carcinoma: resection versus transplantation. *Hepatology*.

[22] Pelletier SJ, Fu S, Thyagarajan V (2009). An intention-to-treat analysis of liver transplantation for hepatocellular carcinoma using organ procurement transplant network data. *Liver Transplantation*.

[23] Yao FY, Ferrell L, Bass NM (2001). Liver transplantation for hepatocellular carcinoma: expansion of the tumor size limits does not adversely impact survival. *Hepatology*.

[24] Onaca N, Davis GL, Goldstein RM, Jennings LW, Klintmalm GB (2007). Expanded criteria for liver transplantation in patients with hepatocellular carcinoma: a report from the international registry of hepatic tumors in liver transplantation. *Liver Transplantation*.

[25] Duffy JP, Vardanian A, Benjamin E (2007). Liver transplantation criteria for hepatocellular carcinoma should be expanded: a 22-year experience with 467 patients at UCLA. *Annals of Surgery*.

[26] Decaens T, Roudot-Thoraval F, Hadni-Bresson S (2006). Impact of UCSF criteria according to pre- and post-OLT tumor features: analysis of 479 patients listed for HCC with a short waiting time. *Liver Transplantation*.

[27] Zavaglia C, De Carlis L, Alberti AB (2005). Predictors of long-term survival after liver transplantation for hepatocellular carcinoma. *American Journal of Gastroenterology*.

[28] Macaron C, Hanouneh IA, Lopez R, Aucejo F, Zein NN (2010). Total tumor volume predicts recurrence of hepatocellular carcinoma after liver transplantation in patients beyond Milan or UCSF criteria. *Transplantation Proceedings*.

[29] Cescon M, Ravaioli M, Grazi GL (2010). Prognostic factors for tumor recurrence after a 12-year, single-center experience of liver transplantations in patients with hepatocellular carcinoma. *Journal of Transplantation*.

[30] Todo S, Furukawa H (2004). Living donor liver transplantation for adult patients with hepatocellular carcinoma: experience in Japan. *Annals of Surgery*.

[31] Kaihara S, Kiuchi T, Ueda M (2003). Living-donor liver transplantation for hepatocellular carcinoma. *Transplantation*.

[32] Gondolesi GE, Roayaie S, Muñoz L (2004). Adult living donor liver transplantation for patients with hepatocellular carcinoma: extending UNOS priority criteria. *Annals of Surgery*.

[33] (2000). American society of transplant surgeons’ position paper on adult-to-adult living donor liver transplantation. *Liver Transplantation*.

[34] Garcia-Retortillo M, Forns X, Llovet JM (2004). Hepatitis C recurrence is more severe after living donor compared to cadaveric liver transplantation. *Hepatology*.

[35] Iwatsuki S, Starzl TE (1988). Personal experience with 411 hepatic resections. *Annals of Surgery*.

[36] Poon RT, Fan ST, Lo CM (2001). Improving survival results after resection of hepatocellular carcinoma: a prospective study of 377 patients over 10 years. *Annals of Surgery*.

[37] Moriguchi M, Takayama T, Higaki T Early cancer-related death after resection of hepatocellular carcinoma.

[38] Ishii H, Furuse J, Kinoshita T (2008). Hepatectomy for hepatocellular carcinoma patients who meet the Milan criteria. *Hepato-Gastroenterology*.

[39] Hung HH, Chiou YY, Hsia CY (2011). Survival rates are comparable after radiofrequency ablation or surgery in patients with small hepatocellular carcinomas. *Clinical Gastroenterology and Hepatology*.

[40] Yamakado K, Nakatsuka A, Takaki H (2008). Early-stage hepatocellular carcinoma: radiofrequency ablation combined with chemoembolization versus hepatectomy. *Radiology*.

[41] Canter RJ, Patel SA, Kennedy T Comparative analysis of outcome in patients with hepatocellular carcinoma exceeding the milan criteria treated with liver transplantation versus partial hepatectomy.

[42] Imamura H, Matsuyama Y, Tanaka E (2003). Risk factors contributing to early and late phase intrahepatic recurrence of hepatocellular carcinoma after hepatectomy. *Journal of Hepatology*.

[43] Wei AC, Tung-Ping Poon R, Fan ST, Wong J (2003). Risk factors for perioperative morbidity and mortality after extended hepatectomy for hepatocellular carcinoma. *British Journal of Surgery*.

[44] Ruzzenente A, Capra F, Pachera S (2009). Is liver resection justified in advanced hepatocellular carcinoma? Results of an observational study in 464 patients. *Journal of Gastrointestinal Surgery*.

[45] Regimbeau JM, Abdalla EK, Vauthey JN (2004). Risk factors for early death due to recurrence after liver resection for hepatocellular carcinoma: results of a multicenter study. *Journal of Surgical Oncology*.

[46] Shi M, Guo RP, Lin XJ (2007). Partial hepatectomy with wide versus narrow resection margin for solitary hepatocellular carcinoma: a prospective randomized trial. *Annals of Surgery*.

[47] Kao WY, Su CW, Chau GY, Lui WY, Wu CW, Wu JC (2011). A comparison of prognosis between patients with hepatitis B and C virus-related hepatocellular carcinoma undergoing resection surgery. *World Journal of Surgery*.

[48] Farges O, Belghiti J, Kianmanesh R (2003). Portal vein embolization before right hepatectomy: prospective clinical trial. *Annals of Surgery*.

[49] Abulkhir A, Limongelli P, Healey AJ (2008). Preoperative portal vein embolization for major liver resection: a meta-analysis. *Annals of Surgery*.

[50] Palavecino M, Chun YS, Madoff DC (2009). Major hepatic resection for hepatocellular carcinoma with or without portal vein embolization: perioperative outcome and survival. *Surgery*.

[51] Kubota K, Makuuchi M, Kusaka K (1997). Measurement of liver volume and hepatic functional reserve as a guide to decision-making in resectional surgery for hepatic tumors. *Hepatology*.

[52] Ogata S, Belghiti J, Farges O, Varma D, Sibert A, Vilgrain V (2006). Sequential arterial and portal vein embolizations before right hepatectomy in patients with cirrhosis and hepatocellular carcinoma. *British Journal of Surgery*.

[53] Jarnagin W, Chapman WC, Curley S (2010). Surgical treatment of hepatocellular carcinoma: expert consensus statement. *HPB*.

[54] Mulier S, Mulier P, Ni Y (2002). Complications of radiofrequency coagulation of liver tumours. *British Journal of Surgery*.

[55] Rhim H, Yoon KH, Lee JM (2003). Major complications after radio-frequency thermal ablation of hepatic tumors: spectrum of imaging findings. *Radiographics*.

[56] Cho YK, Kim JK, Kim MY, Rhim H, Han JK (2009). Systematic review of randomized trials for hepatocellular carcinoma treated with percutaneous ablation therapies. *Hepatology*.

[57] Mulier S, Ni Y, Jamart J, Ruers T, Marchal G, Michel L (2005). Local recurrence after hepatic radiofrequency coagulation: multivariate meta-analysis and review of contributing factors. *Annals of Surgery*.

[58] Chen MS, Li JQ, Zheng Y (2006). A prospective randomized trial comparing percutaneous local ablative therapy and partial hepatectomy for small hepatocellular carcinoma. *Annals of Surgery*.

[59] Livraghi T, Meloni F, Di Stasi M (2008). Sustained complete response and complications rates after radiofrequency ablation of very early hepatocellular carcinoma in cirrhosis: is resection still the treatment of choice?. *Hepatology*.

[60] N’Kontchou G, Mahamoudi A, Aout M (2009). Radiofrequency ablation of hepatocellular carcinoma: long-term results and prognostic factors in 235 Western patients with cirrhosis. *Hepatology*.

[61] Huang J, Hernandez-Alejandro R, Croome KP (2011). Radiofrequency ablation versus surgical resection for hepatocellular carcinoma in childs a cirrhotics-a retrospective study of 1,061 cases. *Journal of Gastrointestinal Surgery*.

[62] Peng ZW, Zhang YJ, Chen MS, Lin XJ, Liang HH, Shi M (2010). Radiofrequency ablation as first-line treatment for small solitary hepatocellular carcinoma: long-term results. *European Journal of Surgical Oncology*.

[63] Huang J, Yan L, Cheng Z (2010). A randomized trial comparing radiofrequency ablation and surgical resection for HCC conforming to the Milan criteria. *Annals of Surgery*.

[64] Lencioni R (2010). Loco-regional treatment of hepatocellular carcinoma in the era of molecular targeted therapies. *Oncology*.

[65] Yao FY, Hirose R, LaBerge JM (2005). A prospective study on downstaging of hepatocellular carcinoma prior to liver transplantation. *Liver Transplantation*.

[66] Roayaie S, Frischer JS, Emre SH (2002). Long-term results with multimodal adjuvant therapy and liver transplantation for the treatment of hepatocellular carcinomas larger than 5 centimeters. *Annals of Surgery*.

[67] Yao FY, Kerlan RK, Hirose R (2008). Excellent outcome following down-staging of hepatocellular carcinoma prior to liver transplantation: an intention-to-treat analysis. *Hepatology*.

[68] Porrett PM, Peterman H, Rosen M (2006). Lack of benefit of pre-transplant locoregional hepatic therapy for hepatocellular cancer in the current MELD era. *Liver Transplantation*.

